# Nutritional Status and Feeding Behavior of Children with Autism Spectrum Disorder in the Middle East and North Africa Region: A Systematic Review

**DOI:** 10.3390/nu15030711

**Published:** 2023-01-30

**Authors:** Monia Kittana, Asma Ahmadani, Keith E. Williams, Amita Attlee

**Affiliations:** 1 Department of Nutrition and Health, College of Medicine and Health Sciences, United Arab Emirates University, Al Ain P.O. Box 15551, United Arab Emirates; 2 Department of Pediatrics, Penn State Hershey Medical Center, Penn State College of Medicine, Hershey, PA 17033, USA

**Keywords:** autism spectrum disorder, feeding, nutrition, anthropometrics, Middle East, North Africa

## Abstract

Autism spectrum disorder (ASD) in children is associated with increased risks of overweight/obesity and underweight, altered nutrient profile, and abnormal feeding behaviors. This systematic review aimed to elucidate the literature on the nutritional status of children with ASD in the Middle East North Africa (MENA) region, by providing a summary and assessment of the body of evidence. A systematic review of English and Arabic publications up to November 2020 was conducted of five databases in addition to the grey literature, which include a nutrition-related parameter, from both experimental and observational study designs. Children with ASD (ASD-C) between 2 and 19 years in the MENA Region were the target population. For risk of bias, the Academy of Nutrition and Dietetics’ Quality Criteria Checklist (QCC) was adopted. The number of published articles was grossly limited. Forty-three articles were included, of which only four articles reported a low risk of bias; therefore, the results were interpreted in light of methodological limitations. Both overweight and underweight were common in ASD-C, although not consistently different than typically developing children. Nutrient inadequacies of energy, protein, omega-3, and others; deficiencies in serum iron indicators and calcium, as well as vitamins B12, B9, and D levels; and higher levels of homocysteine and omega-6/omega-3 ratios were reported. Feeding behavior problems were also common in ASD-C. Understanding nutritional requirements and food preferences can guide the planning of the appropriate comprehensive interventions for ASD-C. Various nutritional and behavioral concerns were identified in the included studies; however, they were subject to methodological weaknesses, which limited the generalizability of these results. Future research is warranted that must be directed to finding strong evidence using robust study designs on nutritional status and feeding behaviors of ASD-C, with a particular emphasis on the MENA Region.

## 1. Introduction

Autism spectrum disorder (ASD) is a neurodevelopmental condition characterized by persistent challenges in social interaction, speech, nonverbal communication, and repetitive/restrictive behavior [1]. Data on prevalence from countries in the Middle East and North Africa (MENA) region are scarce, yet the prevalence in Gulf counties ranged from 1.4 to 29 per 10,000 [2], including 4.3 per 10,000 in Bahrain [3], 20.35 per 10,000 in Oman [2], and 1 in 146 children in the United Arab Emirates (UAE) [4]. Evidence for both genetic and environmental factors contributing to ASD risk has been reported [5]. Common environmental risk factors include advanced parental age, cesarean section, suboptimal breastfeeding, prenatal complications, and lead exposure, among others [3,6,7,8]. 

Restrictive and repetitive diets, presence of medical comorbidities, hyperactivity, abnormal feeding practices, and parental dietary beliefs may increase their risk of under-nutrition, which can significantly impact the health-related quality of life [9,10]. ASD-C may also be predisposed to overweight and obesity attributed to side effects of psychotropic medication, sleeping problems, family environment, motor skill difficulties and lower engagement in physical activity [11,12,13], and food selectivity with the preference for energy-dense foods [14]. 

Further, ASD-C are at an increased risk of altered nutrient profiles. Feeding challenges can exacerbate inadequate nutritional status; deficiencies in folic acid, calcium, sodium, potassium, and zinc, as well as vitamins A, B5, B6, C, and D, may occur, which may exert adverse effects on their development [15,16,17,18]. Moreover, specific nutrients, such as vitamin D, may also play a key role in exacerbating symptoms of ASD [19,20]. Moreover, higher serum levels of saturated fatty acids, and lower levels of vitamin E, glutathione, and some polyunsaturated fatty acids, have been reported [3]. 

Early nutrition is critical for neurodevelopment [21]. It is necessary to understand the current trends in nutritional status and feeding behavior in ASD-C in order to provide an insight into the challenges that necessitate early and comprehensive interventions. The objective of this review is to elucidate the available literature regarding the nutritional status and feeding behavior in ASD-C in the MENA Region. 

## 2. Materials and Methods

### 2.1. Eligibility Criteria

Community-based and clinical studies of any study design were included. The target population was children diagnosed with ASD, of both genders, ages 2–19 years, and where possible, the outcomes were compared with controls—typically developing children (TD-C). For an overall assessment of the nutritional status, the outcomes included anthropometric-related data (weight, height, body mass index (BMI), and circumferences), serum-level-related data (protein and micronutrient adequacy indicators, fatty acids levels, and hematology tests), nutrient-intake-related data (energy, macronutrient, and micronutrient intakes), and feeding-behavior-related data (number of meals and snacks, mealtime behavior, and feeding skills). 

Other inclusion criteria were the availability of full-length published articles in either English or Arabic. Articles presented in conferences, magazines, or newspapers were excluded. If the data were reported in more than one publication, the more recent was included. This systematic review protocol was registered at the International Platform of Registered Systematic Review and Meta-analysis Protocols (INPLASY), registration number INPLASY202310066.

### 2.2. Search Strategy

Five electronic databases were searched: Cochrane library trials, EBSCO: CINAHL Complete, EBSCO: Academic Search Complete, Medline/PubMed, and Web of Science. Studies from any time range until November 2020 were accepted. The key terms included child-related terms separated by “OR”, AND autism-related terms separated by “OR”, AND nutrition-related and eating behavior-related terms separated by OR, AND country-related terms, all separated by the function “OR”. The search strategy is clarified in Table 1. 

Grey literature sources included the followiing: OpenGrey, Clinicaltrials.gov, Sigma Repository, OAIster, WHO library, and Open Access Theses and Dissertations. The search limiters included searching for the keyword “autism” and using filters (language, country, and disciplines) for refining search results. If the filters were not provided, a manual search was conducted. Google search engine was also used in an attempt to find more results, using a combination of the search strategy defined above. 

### 2.3. Study Selection

**Figure 1:**
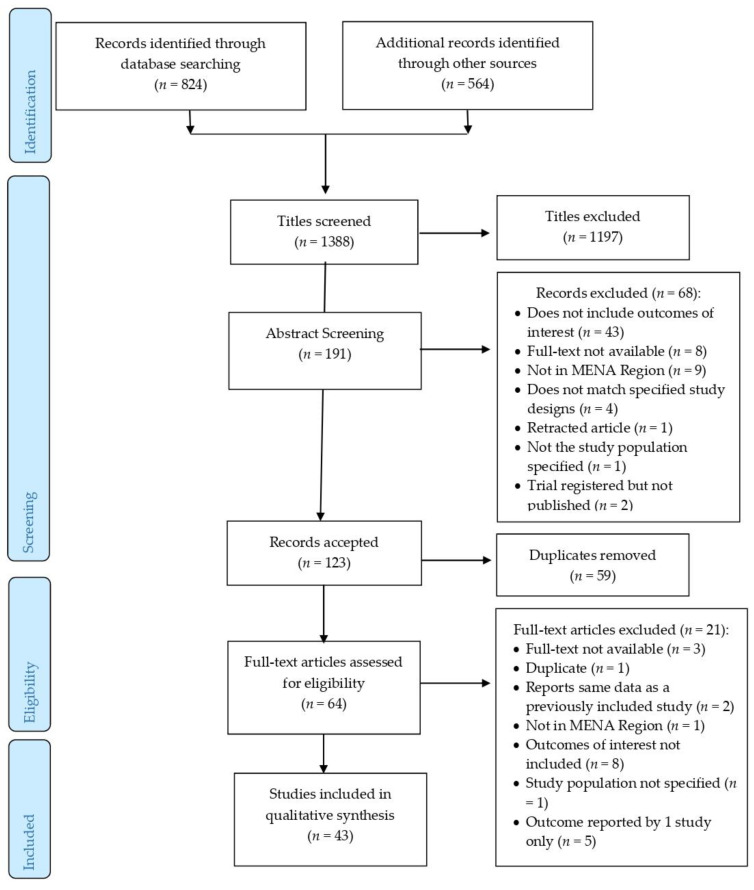
Study selection process.

One reviewer independently completed the study selection process by screening each article against the inclusion criteria of the target population, outcomes of interest, and article type. Figure 1 depicts the PRISMA flowchart for the study selection process [22]. Subsequent to the identification of studies, the titles were screened to include potential studies. Duplicate records were removed in this process. Next, the abstracts were reviewed against the eligibility criteria. Finally, the articles were chosen following the completion of a full-text screening. This process was manual, by saving the potential studies in distinct folders created for each database for each stage. For studies without an available published full-text version, the corresponding author was contacted. If no replies were received within 4 weeks, the article was excluded. 

### 2.4. Data Collection

Data were extracted using a data extraction form designed for the purpose of this review. This form consisted of five main sections. The first section included general information (study characteristics, aim, design, and description of study population (and control group, if applicable)) and was applied to all studies. The rest were applied if the study reported the following outcomes of interest: anthropometric-related data, serum-level-related data, nutrient-intake-related data, or feeding-behavior-related data.

The outcome of interest was the mean and standard deviation (SD) of each value and the difference of means (SD) between ASD-C and TD-C. If an outcome was reported in two or more studies, it was included in the synthesis of results. From experimental studies, only the baseline data of participants were extracted. One author completed the data extraction process and no automation tools were applied in the process. 

Quantitative variables were presented in tables corresponding to each type of nutritional parameter, expressed as the mean (SD) values for ASD-C and TD-C and whether there was a significant difference of means, which allowed for the analysis of the overall trends of nutritional outcomes and assessing the body of evidence. No other methods were applied for further data preparation. For feeding behaviors, all types of data were extracted and organized in a table under the study title, followed by a categorization of recurrent themes (e.g., snacking behaviors, mealtime behaviors, and so on).

### 2.5. Quality Appraisal

Two authors assessed the methodological quality and risk of bias. A third author was consulted in cases of disparities and resolved any disagreements. The Academy of Nutrition and Dietetics’ Quality Criteria Checklist (QCC) was used [23]. Overall, the reviewers rated each study on ten criteria, including the following: clear research questions, selection bias, comparable study groups, study withdrawals, blinding, description of intervention/exposure in detail, clear outcomes, valid and reliable measurements, statistical analysis, conclusions supported by results (bias taken into consideration), and study’s funding/sponsorship [23]. After examining each study’s design and execution, the QCC was used to assign an overall rating. A positive rating is assigned if five or more items are answered with “Yes”, including items 2, 3, 6, and 7, indicating a higher quality study and a less risk of bias. If five or more items are answered with “Yes”, but questions 2, 3, 6, and 7 are answered in a manner that does not indicate that the study is exceptionally strong, a neutral rating is assigned. A negative rating is assigned if six or more questions are answered as “No”. 

## 3. Results

### 3.1. Study Description

Out of 1388 identified records, 43 papers based in 12 MENA countries were included in the final data synthesis. Most studies were from Egypt (*n* = 13) and Saudi Arabia (*n* = 8), followed by Iran (*n* = 6), Oman (*n* = 6), Jordan (*n* = 2), and Qatar (*n* = 2), and one study each from Iraq, Kuwait, Palestine, Syria, Tunisia, and UAE. All were published in English between June 2008 and August 2020. Table 2 presents the studies’ characteristics including the design, aim, cases’ and controls’ characteristics, and recruitment information. Most studies were observational, following either a cross-sectional (*n* = 28) or case-control (*n* = 10) study design, with an exception of four experimental studies, and one study with two phases (cross-sectional design followed by a trial). The sample sizes ranged from 11 to 344 participants, aged between 2 and 19 years, in the selected studies. 

### 3.2. Risk of Bias Assessment

Of the 43 studies, 4 studies had a positive rating, 36 had a neutral rating, and 3 had a negative rating. The quality appraisal results are presented in Supplementary Table S1. Common limitations identified were sampling bias, sample non-representativeness, non-comparable groups, or insufficient description of samples/groupings. Sampling bias was expected owing to the difficulty in recruiting a random sample of ASD-C; therefore, most studies opted for a convenience sample. Observational studies mostly had a lack of blinding for the measurement of outcomes. Other frequent drawbacks included not mentioning methods of handling dropouts or not describing response rates, as seen in studies that obtained a neutral score. Negatively rated studies were restricted with a bias in sample recruitment, incomparable groups, failure to discuss dropout or response rates, conclusions did not consider biases nor study limitations, or possible biases from sponsorships. 

### 3.3. Study Findings

#### 3.3.1. Anthropometric Data

Anthropometric data are presented in Table 3. Regarding height, none of the four studies found a significant difference between ASD-C and controls. Six studies compared the weights of ASD-C with TD-C, two of which reported significantly higher weights in ASD-C [24,55]. Only one out of the five studies that reported BMI showed that ASD-C had a significantly higher BMI than TD-C [57]. The risk of bias in the studies reporting significant outcomes was neutral. Only one study with a positive methodological rating showed similar body weight values between groups with no significant difference. 

Studies used different criteria for defining obesity in children according to BMI categories, including either ≥95th percentile [30,31,35,36,37,38] or ≥97th percentile on the BMI-for-age charts [24,62]. Bener et al., in 2014 and 2017, in their studies of positive methodological quality, compared to other studies reporting this outcome, showed that ASD-C were significantly more likely to fall in the combined underweight and normal weight group compared with TD-C. On the other hand, two studies reported no significant differences [24,31]. One study reported that both overweight and underweight were higher among ASD-C compared with TD-C [48]. In the other eight studies without a control group, four documented that ASD-C were more likely to be of normal weight [35,47,58,60]; however, a higher prevalence of the overweight/obese category in ASD-C was noted in the other four studies [30,62,63,64]. Examining these trends, it should be noted that the selected studies had a neutral risk of bias, except one study with a high risk, which reported a higher prevalence of the overweight/obese category.

#### 3.3.2. Serum-Level Data

Table 4 presents data on hemoglobin (Hb), hematocrit, mean corpuscular volume (MCV), iron status (serum iron or ferritin levels), vitamin B12, and folate. Serum iron was significantly lower in ASD-C, based on two studies with positive and neutral methodological quality, respectively [38,55]. Additionally, Bener et al. (2017) reported significantly lower ferritin levels reflecting storage depletion, concurrent to the reduced serum iron in ASD-C. Al-Ali et al. (2014) also supported low ferritin levels in ASD-C, though not significantly different from TD-C.

A higher prevalence of anemia was evident in ASD-C (37.5%) compared with in TD-C (7.5%) [27]. Hb and hematocrit levels were reported to be significantly lower in two out of three studies in ASD-C, of neutral and positive methodological quality, respectively [25,38]. Bener et al. (2017) reported significantly lower MCV in ASD-C [38], while Al-Ali et al. (2015) reported a trend (*p* = 0.052) of lower mean MCV compared with TD-C and those with other mental disorders [25]. On the other hand, Al-Farsi et al. (2013a) reported a non-significant difference between ASD-C and TD-C [27], which may be expected as both vitamin B12 and folate levels associated with macrocytic anemia were significantly lower in ASD-C [27]. Two more studies reported significantly lower levels of both vitamins in ASD-C [29,55]. The significantly lower serum vitamin levels were based on three studies of neutral ranking in methodological quality. Significantly increased homocysteine was reported [27,29]. 

Serum-related data also included vitamin D, other minerals, and fatty acids (data presented in Supplementary Table S2 (vitamin D and minerals) and Supplementary Table S3 (fatty acids)). Ten studies reported that ASD-C had significantly lower mean serum vitamin D values than the controls [20,33,34,37,38,40,43,46,53,58], two of which had a positive ranking of methodological quality, while the rest were of neutral ranking. Only one study reported a non-significant difference; however, it is noted that the results for both groups reflect deficient levels [49]. Although Javadfar et al. (2020) did not provide a comparison group, the mean 25-OH-D level in ASD-C was severely deficient [51]. For other minerals, a general trend of lower levels in ASD-C was observed. To elaborate, five out of six studies reported significantly lower calcium levels [37,38,41,53,55], two out of three studies reported significantly lower phosphorus levels [37,38], and three out of four studies reported significantly lower magnesium levels than controls [37,38,55]. Zinc and potassium results were inconsistent [26,37,38,41,50].

Essential fatty acids in three studies (two neutral and one negative ranking in the methodological quality) consistently reported significantly lower levels of linolenic acid (omega-3), linoleic acid (omega-6), arachidonic acid (AA) (omega-6), and docosahexaenoic acid (DHA) (omega-3) than controls [52,59,60]. However, the AA/DHA ratio was significantly higher in ASD-C in three studies [52,59,60], and significantly lower in one [42]. Although the results were inconsistent, there seems to be an imbalance in the omega-6/omega-3 ratio among ASD-C.

#### 3.3.3. Nutrient Intake Data

Significant differences were observed in the nutritional intakes of ASD-C and controls. The data on energy and macronutrient intakes (Table 5) show that lower energy intake was a common observation in ASD-C [28,31,48]. Generally, fat intake corresponded with energy intake, being significantly higher with increased caloric intake in ASD-C [24], and significantly lower with decreased caloric intake [31]. Protein intake was generally significantly lower in ASD-C [31,48,55]. No significant associations were observed with carbohydrate intake [24,31,48,55], and fiber intake results were inconsistent [54,55]. Al-Kindi et al. (2016) reported inadequate fiber intakes in both groups, yet significantly lower in ASD-C than TD-C [31]. Regarding macronutrient distribution of the diet, Aghaeinejad et al. (2013) reported a significantly higher fat intake and significantly lower protein and carbohydrate intakes in ASD than in the control group. Meguid et al. (2017) reported similar distributions, with no significant differences [55]. The studies comparing the macronutrient intake of ASD-C with control groups were all of neutral ranking in methodological quality.

Regarding dietary fat quality (Table 6), significantly lower intakes of omega-3 in ASD-C were reported in two studies of neutral methodological quality [28,48]. Javadfar et al. (2020) did not provide a control group, yet mean intakes of omega-3 were grossly inadequate [51]. Saturated fats and cholesterol intakes were also lower in comparison with controls, consistent with a significantly lower total fat intake [31].

Micronutrient intake data are summarized in Table 7. Lower intakes of vitamin D were observed in ASD-C [31,46,48]; however, only one study reported significant differences with TD-C [31]. Vitamin B12 and folate also showed a trend of lower intakes in ASD-C, and two studies reported significantly lower levels in contrast to TD-C [27,55]. There were inconsistencies in vitamins C and B6 (pyridoxine) results. While one study reported significantly higher intakes of both in ASD-C [55], another found no significant differences with TD-C [31]. Vitamins A, B1, B2, B3, and E and phosphorus did not show any significant differences [31,55]. All of the reviewed studies in this section were of neutral ranking in methodological quality.

#### 3.3.4. Feeding Behavior Data

Seven studies reported outcomes related to feeding and mealtime behaviors of ASD-C, out of which six were of neutral ranking of methodological quality, and one was negative. The variability among studies examining feeding behaviors was high; however, some common observations were noted. For example, regarding added sugar consumption, One study reported that 53.3% of ASD-C consumed soft drinks daily compared with 8.3% of TD-C [48]. ASD-C were more likely to consume sweet snacks including sweets and fruits (*p* = 0.02) [45]. A trend of increased sugar and sweet intakes was also reported [61], where almost all ASD-C (96.7%) consumed soft drinks, ranging from once a day (21.2%), twice a day (33.4%), thrice or more a day (11.9%), or on an irregular basis (30.2%); however, there was no comparison group. Similarly, another study reported 70.9% of children preferred sweet food items (e.g., chocolate, candy, cookies, and Arabic desserts) and that 20.1% of children had only one sweet snack item per day, up to 25.9% children having consumed at least two of them per day [61]. On the other hand, compared with TD-C in one study, ASD-C were reported to have no significant differences in the intakes of sweetened juices, sweets, and fruit intakes [48].

Other snacking behaviors were highlighted in different studies, including a significantly higher frequency of four or more snacks per day in 40% of ASD-C compared with 6.7% of TD-C [25], and three or more snacks per day in 35.8% of ASD-C consumed as compared with 20.5% of TD-C [45]. Further, 7.8% of children consumed three or more snacks/day between meals, while 31.7% consumed two snacks per day and 26.2% consumed one snack per day [61]. 

Regarding preferences, ASD-C were reported to have a high food selectivity for starchy foods [36,39]. Other preferences, such as food color, were highlighted, in which ASD-C were found to have a significant preference for the color ‘red’ compared with the control groups [25]. 

ASD-C also display more frequent eating problems. A higher frequency of eating problems including more limited food options (chicken, eggs, vegetables, and fruits), greater fear of trying new foods, pica, and increased difficulty in transitioning to solid foods were evident in ASD-C than TD-C [39]. Regarding food preferences, it was reported that ASD-C consumed significantly less milk, eggs, fresh vegetables, fish, ghee, butter, olive oil, and fast food [48], and showed a rejection rate of about 40% of total food items, with proteins (meats, fish, poultry, beans, and legumes) being the most often rejected in a different study [36]. Similarly, a third study reported that less than half of the children consumed milk, fish and seafood, fresh/cooked vegetables, and fruits [30]. Food neophobia was commonly observed [39], as 55.4% of children refused the introduction of new foods, and 58.8% of the children reported mood changes if a new food was introduced [30]. In contrast, 69.6% of ASD-C were occasionally willing to try new foods, while only 17.4% were never or rarely willing [36].

Further, ASD-C were more likely to demonstrate troubled mealtime behavior at restaurants than TD-C (*p* = 0.001) [39]. Other troubled mealtime behaviors included never remaining seated until the meal was finished (21.7% of ASD-C) [36], and lack of ability to remain calm during mealtime (15.9% of ASD-C), with a higher likelihood in children with a higher BMI (*p* = 0.026) [30].

## 4. Discussion

Despite the increased prevalence of ASD in the MENA region, as well as the observations of an altered nutritional status in ASD-C, data on this topic from the MENA region remain scarce and inconsistent. To the best of our knowledge, this is the first systematic review on the nutritional status of ASD-C in the MENA region, which included 43 studies on ASD-C of both genders, age ranging between 2.0 and 19 years. Our findings indicate that the available literature is highly limited in terms of the quality of the studies. The outcomes of these studies should be interpreted with caution owing to methodological limitations, especially a high risk of bias in sample selection. There was also a high degree of inconsistency in the results, limiting the ability to report and generalize definitive conclusions. These may be attributed to the use of different assessment tools and geographical variations influencing nutritional outcomes. Furthermore, only a few studies reported each outcome. To elaborate, we included circumferences as an outcome measure of anthropometric data; however, it was not reported in any of the reviewed studies. Yet, significantly higher waist circumference [65,66], waist/hip ratios [65], and waist/height ratios [66] have been reported in ASD-C compared with TD-C in the USA and Spain. 

Anthropometric measurement results indicate that the weight and BMI status of ASD-C were usually similar to TD-C. These were consistent with a previous systematic review of 21 studies from various regions [67]. Nonetheless, some studies do raise concern about the ability of ASD-C to maintain a healthy weight, as they were either at a risk of overweight and/or obesity, as evident in Iran and Egypt [24,57], or underweight and malnutrition, as reported in studies from Oman and Qatar [9,37,38]. Hammouda et al. (2018) reported that both overweight and underweight were more common in ASD-C. These studies also differed in defining the cut-off points for obesity, at either the 95th or 97th percentile according to the BMI-for-age charts. A meta-analysis from different continents confirmed that, although ASD-C are more likely to be of normal body weight (52%), the remaining were more likely to be obese, overweight, or underweight (21.8%, 19.8%, and 6.4% respectively), which was higher than TD-C (11.7%, 16.5%, and 4.9% respectively) [68]. Overweight and obesity rates in ASD-C were also reported to be higher than in TD-C in different regions [69,70,71]. Significantly higher growth hormone levels may contribute to this difference [72], although factors other than the presence of a neurodevelopmental disorder, such as socioeconomic status, parental educational attainment, cultural environment, physical activity, and sleeping habits, can influence the weight status of children [73,74,75,76]. Further, Al-Kindi et al. (2016) reported a significantly lower energy intake, without significant difference in the weights of ASD-C and TD-C, and Meguid et al. (2017) reported no differences in caloric intake, but found that ASD-C were heavier than TD-C. However, Aghaeinejad (2013) observed significantly higher weight, energy, and fat intakes in ASD-C than in TD-C.

Owing to the restrictive eating behavior and the tendency of using elimination diets in ASD-C, nutritional adequacies may be compromised in them [67]. This necessitates appropriate and frequent monitoring and evaluation of the nutritional status of ASD-C owing to increased risk of nutrient deficiencies highlighted in this review. Despite mixed results, lower caloric and protein intakes were more commonly observed in ASD-C. Carbohydrate and fat intakes were inconsistent, although significantly lower omega-3 intakes were reported, which also translated into a lower serum profile of DHA [28]. Meguid et al. (2017) showed increased SFA intakes in all age groups among ASD-C. Nutritional inadequacies in micronutrients, most notably of vitamins D and B12 and folate, were observed, in line with the previous review of global studies on ASD-C [67]. Vitamin D deficiency in ASD-C may be attributed to lower sun exposure [37] or heritable vitamin D deficiency [77]. Moreover, it can also be strongly correlated with ASD severity, suggesting that early vitamin D monitoring and intervention is critical [78]. The deficiency levels of folate and vitamin B12 were also reported along a higher MCV in children (Al-Farsi et al., 2013a) and an increase in homocysteine levels (Ali et al., 2011). Homocysteine is significantly elevated in ASD-C [79] and correlates with ASD symptom severity too, indicating the importance of vitamin B12 and folate in the diets to ASD-C [80]. Furthermore, serum iron, ferritin, Hb, and hematocrit levels were commonly reported concerns, found to be significantly lower in Turkish ASD-C than in controls [81].

A high variability was reported in the status of other micronutrients. Micronutrient levels such as zinc could be geographically dependent. Faber et al. (2009) reported lower serum zinc and copper toxicity in ASD-C in the USA [82], whereas these differences were not significant in Brazil [83] and Ireland [84]. Significantly higher levels of serum potassium were reported [37,41], attributed to the increased extracellular potassium in ASD owing to reduced mitochondrial dysfunction, leading to a lower activity of ion pumps [41]. 

The differences reported in food preferences and mealtime behaviors may also influence the nutritional status. Selective eating and food rejection can lead to limited food intakes, compromising the adequacy of diet in important vitamins, minerals, and essential fatty acids [85]. A meta-analysis of 19 studies from the USA, Europe, and Asia also indicated lower nutrient intakes of protein, calcium, phosphorus, selenium, and omega-3, as well as vitamins B1, B2, B12, E, and D intakes, in ASD-C compared with TD-C [65]. The variability in feeding behavior is high among ASD-C [67]. Attlee et al. (2015) did not report a very high frequency of negative mealtime behavior; however, they reported that all children had inadequacies in at least five of the following nutrients: energy, protein, carbohydrates, fats, fiber, calcium, and iron, as well as vitamins A, C, and D.

Further, these behaviors differ between age groups, implying that age-specific analysis is warranted [86]. Feeding disorders, such as food refusals, limited preferences, and disrupted mealtime behavior, are commonly observed during ages 1 year to 3 years old [87]; reported frequently in ASD-C as sensory processing disorders, including tactile, taste/smell, and visual/auditory sensitivities that exert an influence on feeding behaviors [86]. Similar to on the current review on ASD-C, a high prevalence of added sugar consumption [88,89] and presence of disordered feeding behaviors [90,91,92] were also reported among TD-C in the MENA region. These are influenced by maternal obesity [90], mealtime setting, and food introduction methods following weaning [91]. Most studies in our review were limited for not including a comparison group to assess the feeding behavior in ASD-C. However, studies from other regions highlight that ASD-C do present with significantly more rapid increased feeding difficulties [93] and a higher frequency of neophobia, food selectivity, and emotional under-eating than TD-C [66]. 

One of the strengths of this review was the comprehensiveness of the research questions to summarize the nutritional status of ASD-C. Multiple databases enabled a comprehensive search of relevant evidence available in the MENA region. The search query covered both English and Arabic publications, and the grey literature was extensively searched. However, Persian (Iran’s official language) publications were not included. Despite including 20 countries in the search strategy of this review, there is a gross scarcity of available evidence in the MENA region, coupled with high variability among studies, and high heterogeneity in the outcomes discussed. Given the existent inconsistencies and the low methodological quality, further studies evaluating the different outcomes of nutritional status are warranted.

Most studies did not establish correlations between the various outcome measures used for assessing the nutritional status. For example, dietary assessments should be accompanied with biochemical assessments, especially for nutrients of high concern in this region. Dietary assessments should also be analyzed in coherence with mealtime behaviors and dietary preferences, given the high variability among ASD-C. In our results, there was a high variation in weight status, which did not always correlate with the dietary intake. Therefore, other socio-ecological and lifestyle factors should be considered. Overall, there is a need to adopt a comprehensive nutritional assessment approach of a combination of anthropometric, biochemical, clinical, and dietary methods to indicate the nutritional status of ASD-C through appropriate outcome measures. 

## 5. Conclusions

Adapting an appropriate diet for ASD-C is critical as part of the conventional therapy plan because of its influence on disease severity itself; however, this is ideally tailored based on the individual’s nutritional requirements and food preferences. In our review, we identified 43 studies from 12 MENA countries, and noted the prevalence of the triple burden of malnutrition, both higher weight and BMI status and undernutrition, and micronutrients’ deficiencies in serum iron indicators and calcium, as well as vitamins B12, B9, and D, and higher levels of homocysteine and omega-6/omega-3 ratios in ASD-C in the MENA region. Lower intakes of protein and omega-3 fatty acids were also common. A high frequency of mealtime problems; disordered eating; and certain food selectivity behaviors, especially increased intakes of sweet food items and starchy foods, as well as decreased intakes of eggs, milk, vegetables, proteins (poultry and seafood), and fruits, are reported in ASD-C. Many studies have suffered from methodological weaknesses, which may contribute to the inconsistencies in the outcomes for assessing nutritional status and feeding behaviors in ASD-C in the MENA region. Future research must be directed to bring out strong evidence using robust study designs on nutritional status and feeding behaviors of ASD-C for early diagnosis of nutrition-related health issues that would aid in designing targeted interventions for ASD-C in the MENA region. 

## Figures and Tables

**Table 1:** Search strategy.

Child Terms 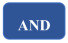	Autism Terms 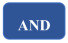	Countries ^†^ 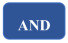	Nutritional Status Terms 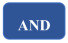	Eating Behavior Terms
Infant Infancy Baby Babies Newborn Toddler Preschool Pre-school Children Child Kindergar * Schoolchild Teen * Youth	Autism Autism, early infantile Autism, infantile Kanner’s syndrome Autistic Autistics disorder	Algeria, Djibouti, Egypt, Bahrain, United Arab Emirates, Qatar, Kuwait, and the Kingdom of Saudi Arabia, Iran, Iraq, Jordan, Lebanon, Libya, Morocco, Oman, Palestine, Sudan, Syria, Tunisia, Tunisia, Yemen, Arab, West Bank, Gaza, Middle East, North Africa, MENA, Middle East North Africa	Nutrition Nutritional status Anthropom * Energy Calorie * Weight * Height * Circumference BMI Body Mass Index Nutrient * Micronutrient Macronutrient Protein Fat * Carbohydrate * Vitamin Mineral Iron Zinc Folate Folic acid	Food intake Ingestion Feeding behavior Diet habits Dietary habits Eating behavior Eating habits Feeding pattern Eating pattern Food habits Food Fussiness Picky eating Meal Snack

Terms in each column were separated by the function ‘OR’. † Each term was included in the search query as follows: (1) If one word only: (“bahrain”[MeSH Terms] OR “bahrain”[All Fields]). (2) If multiple words: (“saudi arabia”[MeSH Terms] OR (“saudi”[All Fields] AND “arabia”[All Fields]) OR “saudi arabia”[All Fields]). * is used in the search term for multiple character searching; to broaden the search by finding word that starts with the same letters.

**Table 2:** Characteristics of studies selected on children with autism spectrum disorder in the MENA region.

#	First Author (Year)	Country	Aim	Study Design	Cases	*n*	Controls	*n*	Recruitment
1	Aghaeinejad (2013) [24]	Iran	Compare nutritional intake with TD-C	Cross-sectional	Male 6–11 years	62	TD-C Male 6–11 years	62	Cases: Elementary schools in Tehran Controls: Elementary schools in Tehran
2	Al-Ali (2014) [25]	Palestine	Investigate iron deficiency association with ASD and compare food selectivity indices	Case-control	Both genders 3–13 years	30	Both genders 3–13 years Group 1: Children with other mental disorders Group 2: TD-C	Group 1: 30 Group 2: 30	Cases: Rehabilitation centers from the North West Bank Control 1: Same rehabilitation centers Control 2: from the general population
3	Al-Bazzaz (2020) [26]	Jordan	Measure fasting levels of glucose, zinc, copper, and zinc/copper ratio, as well as their correlations to the lipid profile	Cross-sectional	Both genders 4–12 years	35	TD-C Both genders 4–12 years	35	Cases: Autism Academy of Jordan Controls: NR
4	Al-Farsi (2011) [9]	Oman	Assess the prevalence of malnutrition indicators	Cross-sectional	Both genders 3–5 years	128	N/A	N/A	Cases: Various social centers and a child psychiatry clinic in Oman Controls: N/A
5	Al-Farsi (2013a) [27]	Oman	Assess the dietary and serum folate and vitamin B12 statuses	Case-control	Both genders 3–5 years	40	TD-C Both genders 3–5 years	40	Cases: Sultan Qaboos University Hospital (SQUH) Controls: Outpatients—Child Health Department at SQUH
6	Al-Farsi (2013b) [28]	Oman	Evaluate the serum levels and dietary intake of docosahexaenoic acid	Case-control	Both genders <5 years	40	TD-C Both genders <5 years	40	Cases: Child and Adolescent Psychiatry—SQUH Controls: Outpatients from the Child Health Department at SQUH
7	Ali (2011) [29]	Oman	Compare serum homo-cysteine, folate, and vitamin B12	Case-control	Both genders 3–5 years	40	TD-C Both genders 3–5 years	40	Cases: NR, Assumed SQUH Controls: Outpatients from the Child Health Department at SQUH
8	Alkazemi (2016) [30]	Kuwait	Evaluate obesity and investigate dietary habits and mealtime behavior	Cross-sectional	Both genders 5–27 years (subgroup: <19 years)	33 (<19 years)	N/A	N/A	Cases: Reach School at Kuwait Center for Autism Controls: N/A
9	Al-Kindi (2016) [31]	Oman	Evaluate the nutritional status through BMI and nutritional intake	Cross-sectional	Both genders 4–13 years	163	TD-C Both genders 4–13 years	212	Cases: SQUH, Developmental Medicine Clinic, Muscat Autism Center, Early Intervention Center for Children with Disabilities, and Al-Waffa Rehabilitation Centers. Controls: Same provinces
10	Al-Kindi (2020) [32]	Oman	Assess food selection criteria, and preferences	Cross-sectional	Both genders 4–13 years	163	TD-C Both genders 4–13 years	212	Same as Al-Kindi 2016
11	Alzghoul (2019) [33]	Jordan	Assess correlation between vitamin D and ASD	Case-control	Male <8 years	83	TD-C Male <8 years	106	Cases: pediatric clinics and healthcare centers Controls: Jordan University Hospital
12	Arastoo (2018) [34]	Iran	Evaluate vitamin D status	Cross-sectional	Both genders 5–12 years	31	TD-C Both genders 5–12 years	31	Cases: Special schools in Ahvaz city Controls: Regular schools in Ahvaz city
13	Ashour (2018) [35]	Saudi Arabia	Investigate the association between dental carries and obesity	Cross-sectional	Females only 6–17 years	41	Other physical or developmental disabilities Females only 6–17 years	234	Cases: various special need schools in Makkah City Controls: Same as cases
14	Attlee (2015) [36]	UAE	Assess the physical status and feeding behavior	Cross-sectional	Both genders 5–16 years	23	N/A	N/A	Cases: Sharjah Autism Center-Sharjah City Controls: N/A
15	Bener (2014) [37]	Qatar	Investigate the association between vitamin D and ASD	Case-control	Both genders <9 years	254	TD-C Both genders <9 years	254	Cases: Pediatrics Clinics and School Health Controls: Primary Health Care centers
16	Bener (2017) [38]	Qatar	Investigate iron and vitamin D deficiency and to assess risk factors	Case-control	Both genders <8 years	308	TD-C Both genders <8 years	308	Cases: Pediatrics Clinic and School Health Controls: Primary Health Care centers
17	Cherif (2018) [39]	Tunisia	Evaluate the frequency and the types of feeding problems	Cross-sectional	Both genders 2–12 years	57	TD-C Both genders Age: NR, but age-matched with cases.	57	Cases: Department of child psychiatry of Sfax Controls: Two Kindergartens from the same area
18	Desoky (2017) [40]	Egypt	Assess thyroid profile, vitamin D levels, and CD5 expression levels, and evaluate correlation with ASD severity	Cross-sectional	Both genders Age range NR. Mean age: 7.03 ± 2.34 years	60	TD-C Both genders Age range NR. Mean age: 7.91 ± 3.21 years	40	Cases: Neuropsychiatric and Pediatric Departments of Qena University Hospital Controls: NR
19	El-Ansary (2010) [41]	Saudi Arabia	Clarify the role of selected ions related to energy metabolism in the deterioration accompanied autism	Cross-sectional	Both genders 3–15 years	30	TD-C Both genders 3–15 years	30	Cases: NR Controls: NR
20	El-Ansary (2011) [42]	Saudi Arabia	Compare the concentrations of essential fatty acids (FAs), polyunsaturated FAs, and phospholipids	Cross-sectional	Both genders 4–12 years	25	TD-C Both genders 4–11 years	16	Cases: Autism Research and Treatment Center Clinic—King Saud University Controls: Well Baby clinic-King Khaled University Hospital
21	El-Ansary (2018) [43]	Saudi Arabia	Determine if there is any relationship between vitamin D levels and ASD presence and severity	Cross-sectional	Male only 3–12 years	28	TD-C Male only Age range NR. Mean age: 7.2 ± 2.14 years	27	Cases: Autism Research and Treatment Center Clinic—King Saud University Controls: siblings of infants from the Well Baby Clinic-King Khalid University Hospital
22	Elbaz (2014) [44]	Egypt	Study plasma essential and non-essential amino acid levels, protein electrophoresis, serum ammonia, and urea	Cross-sectional	Both genders 2–7 years	20	TD-C Both genders 2–7 years	20	Cases: Psychiatric clinic—Children’s Hospital, and Institute of Postgraduate Childhood Studies—Ain Shams University Controls: Pediatrics Outpatient Clinic—Children’s Hospital
23	El-Khatib (2014) [45]	Egypt	Assess the oral health status and behaviors of children	Cross-sectional	Both genders 3–13 years	100	TD-C Both genders 3–13 years	100	Cases: Private and governmental institutions—Alexandria Controls: Private and governmental schools
24	Fahmy (2016) [46]	Egypt	Determine vitamin D dietary intake and sun exposure	Cross-sectional	Both genders 3–15 years	42	TD-C Both genders 3–15 years	40	Cases: Autism clinic—Ain Shams University hospital Controls: Cases siblings
25	Ghodsi (2019) [47]	Iran	Examine carnosine supplementation effects on the advanced glycation end products and the precursors of advanced lipoxidation end products	Randomized controlled trial	ASD-C—with carnosine supplements Both genders 4–14 years	18	ASD-C—without carnosine supplements Both genders 4–14 years	18	Cases: NR Controls: NR
26	Hammouda (2018) [48]	Saudi Arabia	Identify nutritional risk factors that predispose to autism	Case-control	Both genders 2.4–9 years	30	TD-C Both genders 2–9 years	36	Cases: Pediatric psychiatry outpatient clinic and autism day care center—Al-Amal psychiatric hospital. Controls: University and Nabaa AL-Maref Nursery (2–6 years) and researchers’ family members (7–10 years)
27	Hashemzadeh (2015) [49]	Iran	Compare the vitamin D serum levels	Case-control	Both genders 3–12 years	13	TD-C Both genders Age: NR, but matched with cases	14	Cases: Outpatient clinic of Ibn-e-Sina psychiatric hospital Controls: NR
28	Hawari (2020) [50]	Syria	Investigate the levels of lead, manganese, and zinc	Case-control	Group 1: ASD only Both genders 3–12 years Group 2: ASD and ADHD Both genders 3–12 years	Group 1: 31 Group 2: 11	Group 1: ADHD only Both genders 3–12 years Group 2: TD-C Both genders 3–12 years	Group 1: 29 Group 2: 30	Cases: Children Hospital and from related associations Controls: Children Hospital and from related associations
29	Javadfar (2020) [51]	Iran	Evaluate the effect of vitamin D on core symptoms and serum serotonin and IL-6	Randomized controlled trial	ASD-C and vitamin D supplements Both genders 3–13 years	26	ASD-C, but without vitamin D supplements Both genders 3–13 years	26	Cases: Autism clinic of Kermanshah University of Medical Sciences Controls: Autism clinic of Kermanshah University of Medical Sciences
30	Meguid (2008) [52]	Egypt	Estimate free PUFAs in blood and evaluate behavior of children before and after taking fish oil	Before/after trial	Both genders 3–11 years	30	TD-C Both genders 3–11 years	30	Cases: Department of the Children with Special Needs, National Research Center Controls: NR
31	Meguid (2010) [53]	Egypt	Investigate the potential role of vitamin D in autism	Cross-sectional	Gender NR Age range NR. Mean age 5.3 ± 2.8 years	70	TD-C Gender NR Age range NR. Mean age 6.1 ± 1.8 years	42	Cases: Department of the Children with Special Needs, National Research Center Controls: Other clinics at the same facility
32	Meguid (2015) [54]	Egypt	Assess the nutritional status of autistic children	Cross-sectional study	Both genders 3–9 years	80	N/A	N/A	Same as Meguid 2014
33	Meguid (2017) [55]	Egypt	Comparing dietary regimens and habits	Cross-sectional	Both genders 4–6 years	80	TD-C Both genders Age range NR. Age mean 3.7 ± 0.52 years	80	Cases: Department of the Children with Special Needs, National Research Center Controls: NR
34	Meguid (2019) [56]	Egypt	Elucidate the role of zinc supplementation on plasma concentration, gene expression, and cognitive-motor performance	Before/after trial	Both genders 3–8 years	30	N/A	N/A	Same as Meguid 2014
35	Metwally (2018) [57]	Egypt	Assess the concentration of serum BPA and 8-oxodG levels	Cross-sectional	Both genders 5–12 years	49	TD-C Both genders Age range NR. Age mean 5.333 ± 2.279 years	40	Cases: Learning Disability and Neuro-Rehabilitation at Medical Excellence Centre, National Research Centre Controls: NR
36	Mostafa (2012) [58]	Saudi Arabia	Investigate the relationship between vitamin D and anti-myelin-associated glycoprotein auto-antibodies	Cross-sectional	Both genders 5–12 years	50	TD-C Both genders 5–12 years	30	Cases: Autism Research and Treatment Center—King Saud University Controls: Siblings of infants from the Well Baby Clinic—King Khalid University Hospital
37	Mostafa (2015a) [59]	Egypt	Investigate the relationship between serum levels of anti-myelin basic protein auto-antibodies and plasma levels of PUFAs	Cross-sectional	Both genders 4–12 years	80	TD-C Both genders 4–12 years	80	Cases: Pediatric Neuropsychiatric Clinic—Children’s Hospital Controls: Outpatients Clinic—Children’s Hospital,
38	Mostafa (2015b) [60]	Saudi Arabia	Investigate plasma levels of PUFAs and serum carnitine in relation to GI manifestations	Cross-sectional	Both genders 3–10 years	100	TD-C Both genders 3–10 years	80	Same as Mostafa (2012)
39	Murshid (2014) [61]	Saudi Arabia	Report baseline information about the diet, oral hygiene, and dental health of a group of autistic children	Cross-sectional	Both genders 3–14 years	344	N/A	N/A	Cases: 3 autistic rehabilitation centers registered with the Saudi Autistic Society Controls: N/A
40	Saad (2016) [20]	Egypt	Assess vitamin D status compared with controls and the relationship between vitamin D deficiency and autism severity	Cross sectional, followed by a trial	Both genders 3–9 years	122	TD-C Both genders Age range NR. Mean age: 4.88 ± 1.30 years	100	Cases: Assiut university hospitals and five private centers Controls: Assiut university hospitals and siblings of cases
41	Salehi (2014) [62]	Iran	Assess body composition and association of demographic factors, autism severity, and drug therapy	Cross-sectional	Males only 7–14 years	85	N/A	N/A	Cases: Four autism specific schools in Tehran, Controls: N/A
42	Shaaban (2018) [63]	Egypt	Evaluate the efficacy and tolerability of probiotics	prospective, open-label study	Both genders 5–9 years	30	TD-C Both genders Age range not provided, but controls are age-matched	30	Cases: Ain Shams University Hospitals, and The Developmental Pediatric Clinic—National Research Center, Controls: patients’ relatives
43	Wtwt (2015) [64]	Iraq	Assess common feeding problems and nutritional status	Cross-sectional	Both genders Age: >3 years	70	N/A	N/A	Cases: Al Rehma Institute of Autism, Babil Specialized Institute of Autism, and Al Imam Al Husien Institute Controls: N/A

ASD-C: Children with Autism Spectrum Disorder; N/A: Not Applicable; NR: Not Reported; TD-C: Typically Developing Children.

**Table 3:** Anthropometric data of children with autism spectrum disorder and controls in the MENA region.

First Author (Year)	Age (Year)	Height (cm)			Weight (kg)			BMI (kg/m2)/BMI z-Score			BMI Categories n (%)								
											UWT		NWT		OWT		Obese		*p*
		A	C	*p*	A	C	*p*	A	C	*p*	A	C	A	C	A	C	A	C	
Aghaeinejad (2013) [24]	6–11	133.45 ± 8.26	130.57 ± 8.35	0.05	33.14 ± 8.02	30.09 ± 8.18	0.03				6 (10)	8 (13)	29 (47)	31 (50)	7 (11)	13 (21)	20 (32)	10 (16)	0.14
Al-Farsi (2013a) [27]	3–5				15.8 ± 3.1	18.34 ± 2.4													
Alkazemi (2016) [30]	5–19										(6)	N/A	(36)	N/A	O/O (58)	N/A			N/A
Al-Kindi (2016) [31]	4–13				15.4 ± 2.5	15.4 ± 2.6	0.816				47 (28.8)	67 (31.6)	91 (55.8)	126 (59.4)	22 (13.5)	17 (8.0)	3 (1.8)	2 (0.9)	0.301
Arastoo (2018) [34]	5–12	142.35 ± 14.23	137.06 ± 12.52	0.12	42.37 ± 19.55	36.13 ± 12.53	0.14												
Ashour (2018) [35]	6–17										2 (4.8)		23 (56.1)		7 (17.1)		9 (21.9)		N/A
Attlee (2015) [36]	5–16	148.8 ± 20.2	N/A	N/A	78.15 ± 43.7	N/A	N/A	25.5 ± 10.3	N/A	N/A	0 (0)	N/A	6 (26)	N/A	5 (22)	N/A	12 (52)	N/A	N/A
Bener (2014) [37]	<9										U + N 227 (89.4)	U + N 204 (80.3)			22 (8.7)	41 (16.1)	5 (2.0)	9 (3.5)	<0.001
Bener (2017) [38]	<8										U + N 248 (89.4)	U + N 255 (80.3)			39 (8.7)	34 (16.1)	21 (2.0)	15 (3.5)	<0.001
Fahmy (2016) [46]	3–15	120 ± 14.9	123.5 ± 20.1	0.385	27.7 ± 9.3	26.4 ± 12.5	0.613												
Ghodsi (2019) [47]	4–14	134.93	N/A	N/A	31.94	N/A	N/A				6 (16.7)	N/A	23 (63.9)	N/A	7 (19.4)	N/A	0	N/A	N/A
Hammouda (2018) [48]	2.4–9										The prevalence of overweight and underweight was higher among ASD-C (Data not presented)								0.098
Javadfar (2020) [51]	3–13				33.55	N/A	N/A												
Meguid (2015) [54]	3–5	108.32 ± 1.20	N/A	N/A	21.15 ± 1.14	N/A	N/A	F:18.89 ± 1.42/0.4 ± 0.3 M:19.06 ± 2.57/0.95 ± 0.04	N/A	N/A									
Meguid (2015) [54]	6–9	131.08 ± 0.87	N/A	N/A	37.17 ± 1.02	N/A	N/A	F:19.02 ± 1.05/1.6 ± 0.5 M:19.71 ± 1.70/0.9 ± 0.05	N/A	N/A									
Meguid (2017) [55]	4–6	98.5 ± 6.6	97.7 ± 5.7	0.43	16.0 ± 2.3	15.2 ± 1.7	0.02												
Metwally (2018) [57]	5–12							17.713 ± 4.228	15.994 ± 0.691	0.007									
Mostafa (2012) [58]	5–12										All studied subjects had normal body weight (BMI was between the 5th and 85th percentiles based on age and sex)								N/A
Mostafa (2015b) [60]	3–10										All studied subjects had normal body weight (BMI was between the 5th and 85th percentiles based on age and sex)								N/A
Salehi (2014) [62]	7–14	138.56 ± 11.41	N/A	N/A	37.48 + 12.12	N/A	N/A	19.14 + 4.23/NR	N/A	N/A	(9.40)	N/A	(43.50)	N/A	(24.70)	N/A	(22.40)	N/A	N/A
Shaaban (2018) [63]	5–9	122.48 ± 8.27	N/A	N/A	25.91 ± 5.32	N/A	N/A	17.043 ± 1.36/0.80 ± 0.56	N/A	N/A	(0)	N/A	(40)	N/A	(60)	N/A	(0)	N/A	N/A
Wtwt (2015) [64]	3–6										0(0)	N/A	20 (45.5)	N/A	O/O 24 (54.5)	N/A			N/A
Wtwt (2015) [64]	>6										4 (15.4)	N/A	8 (30.8)	N/A	O/O 14 (53.8)	N/A			N/A

A: autism spectrum disorder cases. C: control group. *p*: *p*-value. N/A: not applicable. BMI: body mass index. UWT: underweight. NWT: normal weight. OWT: overweight. U + N: underweight + normal weight. O/O: overweight/obese. F: female. M: male.

**Table 4:** Hematological biomarkers, iron status, vitamin B_12_, folate, and homocysteine data of children with autism spectrum disorder and controls in the MENA region.

**First Author**	**Serum Iron (ug/dL)**			**Vitamin B_12_ (pg/mL)**			**Folate (μg/L)**			**HCY (μmol/L)**		
	**A**	**C**	***p***	**A**	**C**	***p***	**A**	**C**	***p***	**A**	**C**	***p***
Al-Ali (2014) [25]												
Al-Farsi (2013a) [27]				183.6 ± 12.3	341.2 ± 27.4	0.001	2.1 ± 0.3	7.3 ± 0.4	0.001	6.59 ± 0.6	3.92 ± 0.5	0.004
Ali (2011) [29]				191.1 ± 0.9	288.9 ± 1.3	<0.05	1.8 ± 0.4	6.1 ± 0.6	<0.05	20.1 ± 3.3	9.64 ± 2.1	<0.01
Bener (2017) [38]	74.13 ± 21.61	87.59 ± 23.36	0.003									
Meguid (2017) [55]	Lower	Higher	Sig.	Lower	Higher	Sig.	Lower	Higher	Sig.			
**First Author**	**Hb (g/dL)**			**HCT (%)**			**MCV (fL)**			**Ferritin (ng/mL)**		
	**A**	**C**	***p***	**A**	**C**	***p***	**A**	**C**	***p***	**A**	**C**	***p***
Al-Ali (2014) [25]	11.543	MD: 11.960 TD: 12.250	0.016	34.32	MD: 36.453 TD: 35.707	0.016	76.597	MD: 80.243 TD: 78.213	0.052	29.63	MD: 29.513 TD: 35.880	0.316
Al-Farsi (2013a) [27]	11.3 ± 0.7	12.4 ± 0.8	0.43				92 ± 6	83 ± 9	0.21			
Bener (2017) [38]	12.03 ± 2.13	12.86 ± 2.02	<0.001	36.32 ± 2.81	39.07 ± 2.66	<0.001	77.23 ± 5.92	85.24 ± 6.53	<0.001	36.57 ± 5.12	38.49 ± 5.73	<0.001

A: autism spectrum disorder cases. C: control group. *p: p*-value. N/A: not applicable. HCY: homocysteine. Hb: hemoglobin. HCT: hematocrit. MCV: mean corpuscular volume. ug: microgram. pg: picogram. μmol: micromole. g: gram. fL: femtoliter. ng: nanogram. L: liter. dL: deciliter. mL: milliliter. Sig.: significant. MD: mental disorders. TD: typically developing.

**Table 5:** Energy, macronutrient, and fiber intakes data of children with autism spectrum disorder and controls in the MENA region.

**First Author (Year)**	**Energy (kcal/Day)**			**Carbohydrates (g/Day)**			**Protein (g/Day)**			**Fat (g/Day)**			**Fiber (g/Day)**		
	**A**	**C**	***p***	**A**	**C**	***p***	**A**	**C**	***p***	**A**	**C**	***p***	**A**	**C**	***p***
Aghaeinejad (2013) [24]	1926.27 ± 460.07	1647.66 ± 317.14	0.001	240.20 ± 74.59	235.86 ± 48.40	0.7	69.30 ± 21.52	64.08 ± 17.57	0.14	80.48 ± 24.13	52.36 ± 16.07	0.001			
Al-Farsi (2013b) [28]	1323 ± 117.8	1684 ± 101.3	0.001												
Al-Kindi (2016) [31]	1389.6 (56.6)	1594.9 (43.2)	0.005	229.2 (11.1)	248.0 (7.8)	0.16	47.5 (1.8)	55.4 (1.6)	0.002	38.9 (3.3)	47.4 (1.8)	0.015	9.8 (0.7)	12.5 (0.7)	0.017
Hammouda (2018) [48]	1330 ± 541	1576 ± 463	0.051	199 ± 87	234 ± 67	0.076	38 ± 20	48 ± 25	0.091	46 ± 24	54 ± 21	0.159			
Javadfar (2020) [51]	1765.65		NA	246.05		NA	56.65		NA	61.65		NA			
Meguid (2015) (age 3–5) [54]	1490.98 ± 58.51		NA	202.23 ± 7.51		NA	32.77 ± 3.69		NA	61.22 ± 4.61		NA	19.56 ± 2.33		NA
Meguid (2015) (age 6–9) [54]	1875.82 ± 55.32		NA	297.79 ± 32.54		NA	35.58 ± 7.95		NA	60.26 ± 11.80		NA	23.57 ± 2.85		NA
Meguid (2017) [55]	1116.2 ± 271.6	1136.5 ± 269.4	0.317	145.3 ± 22.3	143.0 ± 32.4	0.303	36.6 ± 10.3	39.7 ± 8.7	0.021	43.2 ± 12.8	45.0 ± 11.1	0.233	1.4 ± 0.4	0.715 ± 0.2	0.001

A: autism spectrum disorder cases. C: control group. *p*: *p*-value kcal: kilocalorie. g: grams. N/A: not applicable.

**Table 6:** Dietary fat intake data of ASD-C and controls in the MENA region.

**Author (Year)**	**Omega-3 (g/day)**			**Saturated fat (g/day)**			**MUFA (g/day)**			**PUFA (g/day)**			**Cholesterol (mg/day)**		
	**A**	**C**	***p***	**A**	**C**	***p***	**A**	**C**	***p***	**A**	**C**	***p***	**A**	**C**	***p***
Al-Farsi (2013b) [28]	0.8 ± 0.2	1.2 ± 0.4	0.001												
Al-Kindi (2016) [31]				9.4 (0.9)	14.4 (0.7)	<0.001							122.2 (12.7)	153.3 (10.0)	0.057
Hammouda (2018) [48]	0.029 ± 0.036	0.268 ± 0.498	0.011												
Javadfar (2020) [51]	0.0065		NA												
Meguid (2015) (age 3–5) [54]				21.27 ± 1.45		NA	17.25 ± 1.57		NA	9.35 ± 1.69		NA	289.19 ± 14.75		NA
Meguid (2015) (age 6–9) [54]				29.63 ± 11.30		NA	23.61 ± 2.21		NA	12.35 ± 1.02		NA	258.03 ± 21.05		NA

A: autism spectrum disorder cases. C: control group. *p*: *p*-value. g: grams. mg: milligrams. MUFA: monounsaturated fatty acids. PUFA: polyunsaturated fatty acids. N/A: not applicable.

**Table 7:** Micronutrient intake data of children with autism spectrum disorder and controls in the MENA region.

**First Author (Year)**	**Vitamin A (μg/Day)**			**Riboflavin (mg/Day)**			**Niacin (mg/Day)**			**Pyridoxine (mg/Day)**			**Vitamin C (mg/Day)**			**Vitamin E (mg/Day)**		
	**A**	**C**	***p***	**A**	**C**	***p***	**A**	**C**	***p***	**A**	**C**	***p***	**A**	**C**	***p***	**A**	**C**	***p***
Al-Kindi (2016) [31]	281.6 ± 65.2	306.6 ± 29.5	0.689	1.4 ± 0.2	1.5 ± 0.1	0.615	15.4 ± 0.9	15.2 ± 0.8	0.861	1.2 ± 0.19	1.3 ± 0.1	0.416	47.2 ± 4.6	61.0 ± 4.7	0.057	1.9 ± 0.3	2.2 ± 0.2	0.361
Javadfar (2020) [51]	738.25		NA	1.25		NA	18.15		NA	1.3		NA	119.25		NA	2.4		
Meguid (2015) (age 3–5) [54]	560.19 ± 35.58		NA	0.88 ± 0.10		NA	10.09 ± 5.23		NA	0.91 ± 0.49		NA	39.89 ± 5.41		NA	5.99 ± 0.55 (TR)		
Meguid (2015) (age 6–9) [54]	789.25 ± 32.5		NA	1.28 ± 0.14		NA	13.96 ± 4.35		NA	1.51 ± 0.27		NA	35.99 ± 3.09		NA	7.21 ± 1.01 (TR)		
Meguid (2017) [55]	159.6 ± 30.9 mg	161.8 ± 46.7 mg	0.359	0.47 ± 0.12	0.5 ± 0.13	0.052				0.91 ± 0.16	0.6 ± 0.15	0.001	23.2 ± 6.8	17.7 ± 4.7	0.001			
**First Author (Year)**	**Thiamin (mg/day)**			**Phosphorus (mg/day)**			**Folic acid (μg/day)**			**vitamin B_12_ (μg/day)**			**vitamin D (μg/day)**					
	**A**	**C**	***p***	**A**	**C**	***p***	**A**	**C**	***p***	**A**	**C**	***p***	**A**		**C**		***p***	
Al-Farsi (2013a) [27]							136.3 (5.2)	230.5 (3.7)	0.04	1.3 (0.9)	2.2 (0.8)	0.02						
Al-Kindi (2016) [31]	1.2 (0.1)	1.2 (0.1)	0.913				297.9 (16.1)	297.2 (14.8)	0.977	1.9 (0.7)	1.9 (0.1)	0.993	1.9 (0.2)		3.0 (0.2)		0.0001	
Fahmy (2016) [46]													164.7 ± 71.5 (IU)		177.9 ± 75.9 (IU)		0.42	
Hammouda (2018) [48]							173 ± 128	203 ± 97.9	0.44	0.45 ± 2.27	0.55 ± 1.9	0.365	1.31 ± 1.91		1.60 ± 1.60		0.515	
Javadfar (2020) [51]	1.45		NA				209.2		NA	3.5		NA	0.95 (IU)				NA	
Meguid (2015) (age 3–5) [54]	0.89 ± 0.60		NA	434.28 ± 35.11		NA	195.55 ± 27.21		NA	1.75 ± 1.26		NA	2.34 ± 0.68				NA	
Meguid (2015) (age 6–9) [54]	1.40 ± 1.07		NA	1195.25 ± 68.96		NA	231.03 ± 16.97		NA	2.29 ± 0.97		NA	1.95 ± 0.37				NA	
Meguid (2017) [55]	0.27 ± 0.08	0.28 ± 0.07	0.27	468.2 ± 119.9	469.7 ± 122.7	0.467	197.75 ± 34.22	269.48 ± 20.90	0.001	0.39 ± 0.12	0.85 ± 0.15	0.001						

A: autism spectrum disorder cases. C: control group. *p*: *p*-value. ug: microgram. mg: milligram. N/A: not applicable.

## Data Availability

Data from published articles. No new data were created or analyzed in this study.

## Supplementary Materials

The following supporting information can be downloaded at https://www.mdpi.com/article/10.3390/nu15030711/s1, Table S1: Risk of assessment bias; Table S2: Serum vitamin D, calcium, phosphorus, magnesium, potassium, and zinc levels of children with autism spectrum disorder and controls in the MENA region; Table S3: Serum PUFA levels of children with autism spectrum disorder and controls in the MENA region.
