# Supplementary material for: Nutritional Status and Feeding Behavior of Children with Autism Spectrum Disorder in the Middle East and North Africa Region: A Systematic Review
Source: Nutrients. 2023 Jan 30;15(3):711. doi: 10.3390/nu15030711 (PMC9920501; doi:10.3390/nu15030711)
Supplement: Supplementary file 1 [file nutrients-15-00711-s001.zip › Supplementary Table S1.pdf]

Article title: Nutritional status and feeding behaviors of children with autism spectrum disorder in the Middle East and North Africa Region: A systematic review

Journal name: Nutrients

Supplementary Table S1. *Risk of Assessment Bias*

#	Study	Q1*	Q2*	Q3*	Q4*	Q5*	Q6*	Q7*	Q8*	Q9*	Q10*	Rating
1	Aghaeinejad (2013)	Y	N	Y	N	N	Y	Y	Y	N	N	Neutral
2	Al-Ali (2014)	Y	N	Y	Y	N	Y	Y	Y	N	N	Neutral
3	Al-Farsi (2011)	Y	Y	Y	N	NA	U	Y	Y	Y	U	Neutral
4	Al-Farsi (2013)a	Y	N	Y	N	N	Y	Y	Y	Y	Y	Neutral
5	Al-Farsi (2013)b	Y	N	Y	N	N	Y	Y	Y	N	Y	Neutral
6	Ali (2011)	Y	N	Y	N	N	Y	Y	Y	N	Y	Neutral
7	Alkazemi (2016)	Y	N	Y	N	N	Y	Y	Y	Y	N	Neutral
8	Al-Kindi (2016)	Y	N	Y	N	N	Y	Y	Y	N	N	Neutral
9	Al-Kindi (2020)	Y	Y	Y	N	N	Y	Y	Y	Y	Y	Positive
10	Alzghoul (2019)	Y	N	Y	N	N	Y	Y	Y	Y	Y	Neutral
11	Arastoo (2018)	Y	N	Y	N	N	Y	Y	Y	N	Y	Neutral
12	Ashour (2018)	Y	Y	NA	Y	N	Y	Y	Y	Y	N	Neutral
13	Attlee (2015)	Y	N	NA	N	N	Y	Y	Y	N	Y	Neutral
14	Bener (2014)	Y	Y	Y	Y	N	Y	Y	Y	Y	Y	Positive
15	Bener (2017)	Y	Y	Y	Y	N	Y	Y	Y	Y	Y	Positive
16	Cherif (2008)	Y	N	Y	N	N	Y	Y	Y	N	Y	Neutral
17	Desoky (2017)	Y	N	Y	N	N	Y	Y	Y	Y	Y	Neutral
18	El-Ansary (2010)	Y	N	Y	N	N	Y	Y	Y	N	Y	Neutral
19	El-Ansary (2011)	Y	N	N	N	N	Y	Y	Y	N	Y	Neutral
20	El-Ansary (2018)	Y	N	Y	N	N	Y	Y	Y	Y	Y	Neutral
21	Elbaz (2014)	Y	N	N	N	N	Y	Y	Y	Y	N	Neutral
22	Al-Bazzaz (2020)	Y	N	Y	N	N	Y	Y	Y	N	N	Neutral
23	El-Khatib (2014)	Y	N	Y	N	N	Y	N	Y	Y	Y	Neutral
24	Fahmy (2016)	Y	N	Y	N	Y	Y	Y	Y	N	Y	Neutral
25	Ghodsi (2019)	Y	N	Y	Y	N	Y	Y	Y	Y	Y	Neutral

#	Study	Q1*	Q2*	Q3*	Q4*	Q5*	Q6*	Q7*	Q8*	Q9*	Q10*	Rating
26	Hammouda (2018)	Y	N	Y	N	N	Y	Y	Y	N	Y	Neutral
27	Hashemzadeh (2015)	Y	N	Y	N	N	Y	Y	Y	N	Y	Neutral
28	Hawari (2020)	Y	N	U	N	N	Y	Y	Y	Y	Y	Neutral
29	Javadfar (2020)	Y	Y	Y	Y	Y	Y	Y	Y	Y	Y	Positive
30	Meguid (2008)	Y	N	U	U	N	Y	Y	N	N	N	Negative
31	Meguid (2010)	Y	N	N	N	N	Y	Y	Y	Y	Y	Neutral
32	Meguid (2015)	Y	N	NA	N	N	Y	Y	Y	N	Y	Neutral
33	Meguid (2017)	Y	N	Y	N	N	Y	Y	Y	N	Y	Neutral
34	Meguid (2019)	Y	N	NA	N	N	Y	Y	Y	N	Y	Neutral
35	Metwally (2018)	Y	N	Y	N	N	Y	Y	Y	N	N	Neutral
36	Mostafa (2012)	Y	N	Y	N	N	Y	Y	Y	Y	Y	Neutral
37	Mostafa (2015)	Y	N	Y	N	N	Y	Y	Y	Y	Y	Neutral
38	Mostafa (2015) (2)	Y	N	Y	N	N	Y	Y	Y	Y	Y	Neutral
39	Murshid (2014)	Y	N	NA	N	N	Y	N	Y	Y	N	Negative
40	Saad (2016)	Y	N	U	Y	N	Y	Y	Y	Y	Y	Neutral
41	Salehi (2014)	Y	Y	NA	N	N	Y	Y	Y	Y	Y	Neutral
42	Shaaban (2018)	Y	N	NA	N	N	Y	Y	Y	Y	Y	Neutral
43	Wtwt (2015)	Y	N	NA	U	NA	Y	U	Y	N	N	Negative

Y=Yes, N=No, NA=Not Applicable, U=Unclear
