# Supplementary material for: Nutritional Status and Feeding Behavior of Children with Autism Spectrum Disorder in the Middle East and North Africa Region: A Systematic Review
Source: Nutrients. 2023 Jan 30;15(3):711. doi: 10.3390/nu15030711 (PMC9920501; doi:10.3390/nu15030711)
Supplement: Supplementary file 1 [file nutrients-15-00711-s001.zip › Supplementary Table S2.pdf]

Article title: Nutritional status and feeding behaviors of children with autism spectrum disorder in the Middle East and North Africa

Region: A systematic review

Journal name: Nutrients

Supplementary Table S2: *Serum vitamin D, calcium, phosphorus, magnesium, potassium and zinc levels of children with autism spectrum disorder and controls in MENA region*

Author (Year)	25-OH D (ng/ml)			Calcium (mmol/l)			Phosphorous (mmol/L)			Magnesium (mmol/L)			Potassium (mmol/L)			Zinc (μg/dl)		
	A	C	P	A	C	P	A	C	P	A	C	P	A	C	P	A	C	P
**Alzghoul (2019)**	23.4	37.5	0.00001	9.6 [-]	10 [-]	●	●	●	●	●	●	●	●	●	●	●	●	●
**Arastoo (2018)**	9.03 ± 4.14 ng/mg	15.25 ± 7.89 ng/mg	< 0.001	●	●	●	●	●	●	●	●	●	●	●	●	●	●	●
**Bener (2014)**	18.39±8.2	21.59±8.4	0.004	2.12± 0.1	2.37±0.1	<0.001	1.36 ± 0.3	1.53±0.3	0.01	0.83± 0.1	0.88±0.10	<0.001	5.58± 0.2	4.51± 0.7	<0.001	●	●	●
**Bener (2017)**	18.79±8.35	22.18 ± 9.00	0.004	2.09±0.12	2.39±0.14	<0.001	1.362 ±0.31	1.553± 0.28	<0.001	0.826 ±0.07	0.884±0.06	<0.001	4.252 ±0.50	4.575± 0.55	<0.001	●	●	●
**Desoky (2017)**	18.63 + 10.8	45.9 +8.85	<0.001	●	●	●	●	●	●	●	●	●	●	●	●	●	●	●
**El-Ansary (2010)**	●	●	●	4.84±1.25	11.67±2.04	<0.05	●	●	●	2.04± 0.49	1.83±0.34	NS	8.55± 5.76	4.99±2.12	<0.05	●	●	●
**El-Ansary (2018)**	95.63 ± 26.63	140.43 ± 17.68	0.001	●	●	●	●	●	●	●	●	●	●	●	●	●	●	●
**Al-Bazzaz (2020)**	●	●	●	●	●	●	●	●	●	●	●	●	●	●	●	84.34 ± 18.28	95.89 ± 10.91	<0.05
**Fahmy (2016)**	Median: 46.5 nmol/l	Median: 70.89 nmol/l	<0.001*	9±1.3 mg/dl	9.2±1 mg/dl	0.468	4.9±0.4 mg/dl	4.9±0.5 mg/dl	0.475	●	●	●	●	●	●	●	●	●
**Hashemzadeh (2014)**	Median: 13.0 [-]	Median: 12.0 [-]	0.35	●	●	●	●	●	●	●	●	●	●	●	●	●	●	●

Author (Year)	25-OH D (ng/ml)			Calcium (mmol/l)			Phosphorous (mmol/L)			Magnesium (mmol/L)			Potassium (mmol/L)			Zinc (µg/dl)		
	A	C	P	A	C	P	A	C	P	A	C	P	A	C	P	A	C	P
**Hawari (2020)**	●	●	●	●	●	●	●	●	●	●	●	x●	●	●	●	ASD: 84.48 ± 15.99<br>ASD with ADHD: 83 ± 18.75	TD: 79.97 ± 13.72<br>ADHD: 83.45 ± 13.42	NS
**Javadfar (2020)**	9.515	●	N/A	●	●	●	●	●	●	●	●	●	●	●	●	●	●	●
**Meguid (2010)**	28.5 ± 16.4	40.1 ± 11.8	<0.00001	8.9±0.8 mg/dl	9.5± 0.8 mg/dl	<0.0001	●	●	●	●	●	●	●	●	●	●	●	●
**Meguid (2017)**	●	●	●	Lower	Higher	Sig.	●	●	●	Lower	Higher	Sig.	●	●	●	●	●	●
**Mostafa (2012)**	Median: 18.5	Median : 33	P < 0.001	●	●	●	●	●	●	●	●	●	●	●	●	●	●	●
**Saad (2016)**	18.02 + 8.75	42.51 + 9.48	≤0.0001	●	●	●	●	●	●	●	●	●	●	●	●	●	●	●

A: Autism cases. C: Control group. P: P-value. ●: Not Reported. N/A: Not Applicable. IQR: Interquartile Range. Sig.: Significant. [-]: No unit

* Unit is as shown in the top row unless indicated otherwise in the cells.
