# Supplementary material for: Nutritional Status and Feeding Behavior of Children with Autism Spectrum Disorder in the Middle East and North Africa Region: A Systematic Review
Source: Nutrients. 2023 Jan 30;15(3):711. doi: 10.3390/nu15030711 (PMC9920501; doi:10.3390/nu15030711)
Supplement: Supplementary file 1 [file nutrients-15-00711-s001.zip › Supplementary Table S3.pdf]

Article title: Nutritional status and feeding behaviors of children with autism spectrum disorder in the Middle East and North Africa

Region: A systematic review

Journal name: Nutrients

Supplementary Table S3: *Serum PUFA levels of children with autism spectrum disorder and controls in MENA region*

Author (Year)	DHA (µg/mL)			Linolenic (µg/mL)			Linoleic (µg/mL)			Arachidonic (µg/mL)			AA/DHA		
	A	C	P	A	C	P	A	C	P	A	C	P	A	C	P
Al-Farsi (2013b)	4.1 ± 0.3	8.7 ± 1.1	0.001	●	●	●	●	●	●	●	●	●	●	●	●
El-Ansary (2011)	●	●	●	●	●	●	●	●	●	●	●	●	0.33 ± 0.14	0.81 ± 0.17	0.000
Meguid (2008)	0.95±0.2	2.85±0.65	<0.0001	0.86±0.44	3.2±0.72	<0.0001	1.75±0.46	2.77±0.64	<0.0001	2.5±0.5	4.65±0.5	<0.0001	2.77 ±0.84	1.71 ±0.4	<0.0001
Mostafa (2015a)	0.28 ± 0.03 mmol/L	0.98 ± 0.3 mmol/L	<0.001	0.2 ± 0.04 mmol/L	0.43 ± 0.1 mmol/L	<0.001 mmol/L	0.38 ± 0.11 mmol/L	0.58 ± 0.16 mmol/L	0.01	1.6 ± 0.32 mmol/L	2.6 ± 0.9 mmol/L	<0.05	6.3 ± 1.1	3.2 ± 1.2	<0.001
Mostafa (2015b)	Median: 0.18	Median: 3.1	<0.001	Median:1.6	Median: 3.3	<0.01	Median: 1.3	Median: 3.2	<0.05	Median: 1.7	Median: 4.2	<0.01	3 ± 2.4	1.8 ± 1.4	<0.01

A: Autism cases. C: Control group. P: P-value. ug: Microgram. mL: milliliter ●: Not Reported. N/A: Not Applicable. DHA: Docosahexaenoic Acid. AA: Arachidonic Acid.

A: Autism cases. C: Control group. P: P-value. ●: Not Reported. N/A: Not Applicable. IQR: Interquartile Range. Sig.: Significant. [-]: No unit

* Unit is as shown in the top row unless indicated otherwise in the cells.
